# Neuroform atlas versus LVIS–assisted coil embolization of wide-neck intracranial aneurysms: a retrospective cohort study

**DOI:** 10.3389/fneur.2025.1651702

**Published:** 2025-10-08

**Authors:** Yang Zhang, Shengli Shen, Zhangzheng Liao, Hongzhou Duan

**Affiliations:** Department of Neurosurgery, Peking University First Hospital, Beijing, China

**Keywords:** aneurysm, embolization, stent, endovascular treatment, wide necked

## Abstract

**Background:**

Currently, Neuroform Atlas stent and LVIS stent are widely used in assisting coil embolization of wide-necked intracranial aneurysms (WNIAs), but direct comparisons of their safety and effectiveness are rare. This study aims to compare the embolization effect, complications, and prognosis of Neuroform Atlas stent and LVIS stent in assisting coil embolization of WNIAs.

**Methods:**

We retrospectively analyzed 60 patients who underwent coil embolization of WNIAs with the aid of Neuroform Atlas stent or LVIS stent from July 2017 to January 2024. They were divided into Neuroform Atlas group (*n* = 26) and LVIS group (*n* = 34). The patients’ baseline data, immediate effects, perioperative complications, and follow-up data were collected and compared.

**Results:**

A total of 60 patients with 61 WNIAs underwent stent-assisted coiling with deployment of Atlas (*n* = 29) or LVIS (*n* = 34). The immediate complete obliteration (Class I of Modified Raymond-Roy Classification, MRRC) rate of aneurysm was 81.5% (22/27) in the Atlas group and 73.5% (25/34) in the LVIS group. The rates of intraoperative and postoperative ischemic and hemorrhagic complications were 0 and 5.9% (2/34) per patient in the Atlas group and LVIS group, respectively. Follow up digital subtraction angiography (DSA) showed that 88% (22/25) of aneurysms in Atlas group and 87.5% (28/32) of aneurysms in LVIS group were completely occluded with MRRC grade 1. There was no significant difference in the degree of occlusion (*p* = 0.955) or modified Rankin Scale (mRS) score (*p* = 0.438) between the two groups at follow-up.

**Conclusion:**

In our single-center retrospective exploratory study, both Neuroform Atlas stent and LVIS stent seem to perform good safety and efficacy in assisting coil embolization of WNIAs, however, further multicenter randomized controlled studies with larger patient series and a longer follow up period will be helpful in elucidation of both the efficacy and the longevity.

## 1 Introduction

With the development of interventional devices, endovascular treatment has become one of the main therapies of intracranial aneurysms (1). A wide-necked intracranial aneurysm (WNIA) is defined as an aneurysm with a neck width ≥4.0 mm or a maximum aneurysm dome-diameter-to-neck-width ratio < 2, or both (2). As the neck is relatively wide, the coil is easily displaced into the parent artery or the distal end when treated with conventional coil embolization. In 1997, Higashida used an intravascular stent and coils in the treatment of a ruptured fusiform aneurysm of the basilar artery (3). Compared with the conventional endovascular treatment method, the stent can form a barrier at the neck of the intracranial aneurysm, preventing the coil from falling out through the wide aneurysm neck. Previous studies have confirmed that stent-assisted coiling (SAC) technique yields favorable long-term anatomical results in the treatment of WNIAs.

Currently, the vascular stents used for SAC mainly include laser-cut stents such as Neuroform Atlas (Stryker, USA) and Enterprise (Codman, USA), and braided stents such as LVIS (Microvention, USA) and LEO (Balt, France). These two types of stents differ significantly in mechanical structure and physical properties (4). Neuroform Atlas stent and LVIS stent are commonly used in assisting coil embolization in the treatment of WNIA in Peking University First Hospital. Herein, we retrospectively review the WNIA patients who were previously treated with these two stents in our hospital and analyze their efficacy. We also conducted a literature review to analyze the safety and reliability of these two stents.

## 2 Methods

### 2.1 Patient population

This study retrospectively analyzed patients with intracranial aneurysms treated by one neurointerventional group in the Department of Neurosurgery at Peking University First Hospital from July 2017 to January 2024. Inclusion criteria were:(1) Patients older than 18 years; (2) Patients underwent imaging examinations, such as digital subtraction angiography (DSA), computed tomography angiography (CTA), and magnetic resonance angiography (MRA) to confirm the diagnosis of WNIAs (diameter ≥ 4.0 mm or dome-to-neck ratio < 2); (3) Patients with indications for aneurysm surgery which were the same as reported in literature (1) (ruptured aneurysm or corresponding clinical manifestations, or large aneurysm, irregular shape or sub aneurysm, etc.); (4) Patients or family members agreed to do the neurointerventional operation; (5) Patients were treated with only one kind of stent, namely Neuroform atlas stent or LVIS stent; (6) Patients were able to receive regular follow-up and signed informed consent form. Exclusion criteria were: (1) Patients with a preoperative Hunt-Hess grade of 5. (2) Patients complicated with severe cardiac insufficiency or pulmonary insufficiency, and there were contraindications to anesthesia or surgery; (3) Patients were allergic to contrast agents or metals; (4) Concurrent use of other brands of stents or intrasaccular flow disruptor device (IFDD); (5) There were contraindications related to stent implantation, such as the inability to use antiplatelet due to gastrointestinal bleeding, hematological diseases, etc. (6) Fusiform or pseudoaneurysms; (7) Patients failed to complete at least once follow-up or review.

Finally, 60 patients who underwent coil embolization for WNIAs assisted by the Neuroform Atlas stent or LVIS stent to assist coil embolization of WNIAs were enrolled, of which 8 patients had subarachnoid hemorrhage (SAH). Written informed consent was obtained from all participants. According to the type of stent used for assisting embolization, the patients were divided into the Neuroform Atlas group (*n* = 26) and LVIS group (*n* = 34). The basic data of the patients, such as gender, age, past history (hypertension, coronary heart disease, diabetes, smoking and drinking history), SAH, aneurysm location and size, Hunt-Hess grade at admission, modified Fisher score at admission, immediate intraoperative embolization effect, surgical complications, and follow-up angiography results, were collected and analyzed. The measurement of aneurysms was performed according to the current international common measurement standards (5). The neck of the aneurysm was defined as the line connecting the root of the aneurysm protrusion along the direction of the parent artery, and the diameter of an aneurysm body refers to the distance from the midpoint of the aneurysm neck to the top of the aneurysm. Statistical analyses were performed using SPSS 26.0. This retrospective study was approved by the Medical Ethics Committee at Peking University First Hospital.

### 2.2 Preoperative management

Patients with unruptured intracranial aneurysms received dual antiplatelet therapy (aspirin 100 mg/day and clopidogrel 75 mg/day) for at least 5 days prior to surgery. For ruptured aneurysms, antiplatelet drugs were not routinely used before surgery. Preoperative evaluations included electrocardiogram (ECG), complete blood count, blood biochemical examination, coagulation function tests, chest X-ray, and bilateral lower limb vascular ultrasound. For patients over 60 years old, additional tests such as blood gas analysis and echocardiography were required. The initial diagnosis of aneurysm was made based on head MRA or CTA. Stable patients with ruptured aneurysms typically received endovascular treatment within 24 h of admission, otherwise, the surgery would be postponed until the condition was stable.

### 2.3 Interventional procedures

After general anesthesia, intravenous heparin was administered at 50–100 U/kg to achieve an activated clotting time >200 s. Cerebral DSA was performed first, followed by rotational angiography with 3D reconstructions to display the aneurysm’s location, morphology, size, and orientation. After introducing the guiding catheter, an optimal working view was selected. The stent delivery catheter, guided by a microwire, usually a Traxess 14 (Microvention, USA) or Synchro 14 (Stryker, USA), was placed distal to the aneurysm. The choice of stent delivery catheter depended on the stent used: a Headway 21 or Headway 17 microcatheter (Microvention, USA) for an LVIS or LVIS Jr. stent, and an SL 10 (Stryker, USA) or Echlon 10 (Medtronic, USA) microcatheter for a Neuroform Atlas stent. The embolization microcatheter was shaped according to the preoperative aneurysm morphology and its anatomical relationship with the parent artery. The microcatheter, guided by a microwire, was advanced into the aneurysm sac, and a 3D coil (e.g., ev3 Axium or Prime series) (Medtronic, USA) was deployed. If the coil tended to protrude into the parent artery, an auxiliary stent was placed and deployed through the stent microcatheter. The choice of stent is generally determined by the surgeon based on the shape, locatison, and relationship with the parent artery of the aneurysm. In the Atlas stent group, the appropriate stent size was selected according to the diameter of the parent artery and the width of the aneurysm neck. Sometimes, two Atlas stents forming a cross “Y” shape technique was used in bifurcation WNIA embolization (6). In the LVIS stent group, for aneurysms located in the internal carotid artery or vertebrobasilar artery, the LVIS stent was generally of conventional size (3.5 mm, 4.5 mm or 5.5 mm), and for aneurysms located in the middle cerebral artery, anterior communicating artery, anterior cerebral artery, and posterior cerebral artery, the LVIS Jr. stent (2.5 mm or 3.5 mm) was generally applied. It should be pointed out that for lobulated aneurysms, embolization microcatheter can usually only be placed in one lobe, which will lead to the distribution of coils be often uneven. In this case, after releasing the Atlas stent, we usually shaped the microcatheter and placed it into another lobe of the aneurysm through the mesh of the Atlas stent. The aneurysm was finally densely occluded by zonal filling coils. For patients using LVIS stents, if the aneurysm was lobulated, we usually needed three sets of microcatheter systems. Two sets of microcatheters were placed in different lobes of the aneurysm, and the other set was used to release the stent. The triple catheter technique was not commonly used in the process of LVIS stent assisted embolization, instead, for lobulated aneurysms, semi-jailing technique was more commonly used (7). The LVIS stent was partially deployed approximately one-third to reduce the effective neck size and to prevent distal coil migration. Because the coil delivery catheter is not jailed to the vessel wall, it remains freely maneuverable during the procedure and permit the passive kick-back movement of the microcatheter while decreasing the risk of aneurysm perforation during coil packing, the LVIS stent was fully deployed after achieving the dense coil packing. Then the microcatheter was then withdrawn. Angiography via the guiding catheter was then performed to evaluate the grade of aneurysm occlusion and confirm the parent artery and its branches remain unaffected. A Dyna CT was performed to check that there was no new intracranial hemorrhage or ischemic stroke. Technical success was defined as the stent was deployed across the neck and the aneurysm was densely packed. The occlusion grade of intracranial aneurysm was evaluated based on DSA findings using the Modified Raymond-Roy Classification (MRRC). Class I: complete obliteration; Class II: residual neck; Class IIIa: residual aneurysm with contrast within coil interstices; Class IIIb: residual aneurysm with contrast along aneurysm wall (8).

### 2.4 Postoperative management and follow-up

Patients received routine ECG monitoring, oxygen inhalation and blood pressure control after operation. For the patients with ruptured aneurysms not pretreated with dual antiplatelet drugs and those with intraoperative in-stent thrombosis, a loading dose of tirofiban (0.4 μg/kg/min for 3 min) was administered intravenously after stent release, followed by a maintenance infusion (0.1 μg/kg/min). After operation, the patients were transitioned to dual antiplatelet therapy (tirofiban was discontinued 6 h after loading doses of aspirin 300 mg and clopidogrel 300 mg, followed by aspirin 100 mg/d and clopidogrel 75 mg/d). The patients with hydrocephalus or SAH underwent lumbar puncture and external drainage. After discharge, dual antiplatelet therapy (aspirin 100 mg/d and clopidogrel 75 mg/d) was continued for half a year, followed by a single antiplatelet drug thereafter.

Patients were followed up regularly via phone or outpatient visit every 3 months after discharge. The modified Rankin Scale (mRS) score was assessed 3 months after operation, with a score of 0–2 indicating a favorable outcome and 3–6 indicating a poor outcome. CT, CTA, DSA were performed regularly during follow-up. The first follow-up cerebral DSA was performed 6 months to 1 year after operation, and if the aneurysm was not occluded completely, repeat cerebral angiography was performed annually.

### 2.5 Statistical analysis

All data in this study were statistically analyzed using SPSS26.0 software. Categorical variables were expressed as counts and percentages (%). Continuous variables with a normal distribution were expressed as mean ± standard deviation (X ± SD), and those with a non-normal distribution were presented as median with interquartile range (IQR). Normally distributed continuous variables between groups were analyzed by t test, and non-normally distributed variables were analyzed by nonparametric test (Mann–Whitney U test). The chi-square test was used for categorical variables. The rank sum test (Mann–Whitney U test) was used for ordinal data. Results were considered statistically significant when the *p*-value was < 0.05.

## 3 Results

### 3.1 Baseline characteristics

A total of 60 patients with 61 aneurysms were included in this study. Among the 60 patients, there were 24 males and 36 females with a mean age of 64.6 ± 10.8 years. The Neuroform Atlas group consisted of 26 patients with a total of 27 intracranial aneurysms, treated with 29 Neuroform Atlas stents for assisted coiling embolization. The LVIS group included 34 patients with a total of 34 aneurysms, treated with 34 LVIS (or LVIS Jr) stents. All patients underwent coil embolization using ev3 Axium or Prime series coils. Among all the aneurysms, 54 were located in the anterior circulation and 7 in the posterior circulation. There were no statistically significant differences in baseline characteristics including age, gender, history of hypertension, coronary artery disease, diabetes, smoking history, alcohol consumption history, proportion of aneurysm rupture with SAH, Hunt-Hess grade at admission, location of aneurysm, and modified Fisher score between the two groups (Table 1).

**Table 1:** Baseline characteristics of patients in Atlas and LVIS groups.

Baseline characteristics	Atlas	LVIS	*p* value
N	26 (43.3%)	34 (56.7%)	
Male	11 (42.3%)	13 (38.2%)	0.750
Age (X ± S, years)	67.73 ± 10.49	62.5 ± 11.02	0.068
Hypertension	17 (65.4%)	14 (41.2%)	0.063
Coronary heart disease	5 (19.2%)	6 (17.6%)	0.875
Diabetes	6 (23.1%)	7 (20.6%)	0.817
Smoking	7 (26.9%)	8 (23.5%)	0.764
Drinking	6 (23.1%)	6 (17.6%)	0.602
SAH	2 (7.7%)	6 (17.6%)	0.459
Hunt-Hess scale			0.368
Grade 0	24 (92.3%)	28 (82.4%)	
Grade 1	0 (0%)	0 (0%)	
Grade 2	0 (0%)	1 (2.9%)	
Grade 3	2 (7.7%)	4 (11.8%)	
Grade 4	0 (0%)	1 (2.9%)	
Grade 5	0 (0%)	0 (0%)	
Modified Fisher scale			0.521
Grade 0	23 (88.5%)	28 (82.4%)	
Grade 1	1 (3.8%)	0 (0%)	
Grade 2	0 (0%)	0 (0%)	
Grade 3	1 (3.8%)	2 (5.8%)	
Grade 4	1 (3.8%)	3 (8.8%)	
Aneurysm location			0.628
Anterior circulation	25 (92.6%)	29 (85.3%)	
Posterior circulation	2 (7.4%)	5 (14.7%)	
Aneurysm size			
size<5 mm	13 (48.1%)	11 (32.4%)	0.294
5 mm ≤ size<15 mm	13 (48.1%)	22 (64.7%)	
15 mm ≤ size<25 mm	1 (3.7%)	1 (2.9%)	
Size≥25 mm	0 (0%)	0 (0%)	

### 3.2 Immediate intraoperative outcomes

**Figure 1:**
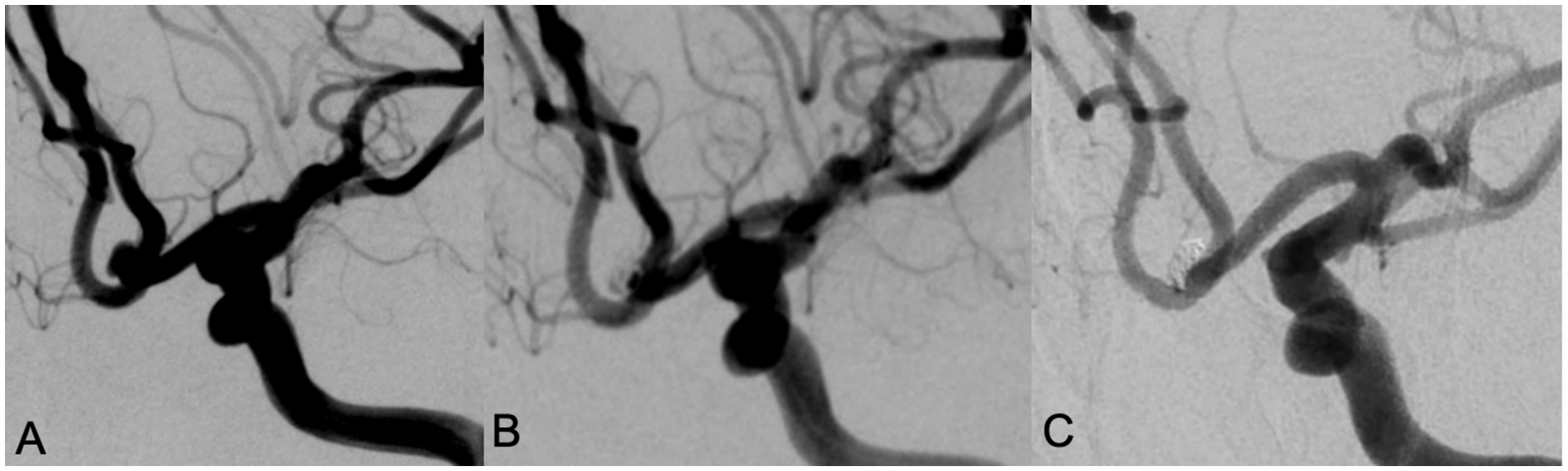
A 78 year old female with intermittent headache and left lower limb weakness for 9 days was admitted, DSA examination showed an anterior communicating artery aneurysm with a wide neck **(A)**. Neuroform Atlas stent assisted coiling embolization was performed. Immediate angiography after embolization showed the aneurysm was almost occluded with MRRC grade 2 **(B)**. Angiography performed in 12 months’ follow-up showed that the aneurysm was occluded well with MRRC grade 1 **(C)**.

**Figure 2:**
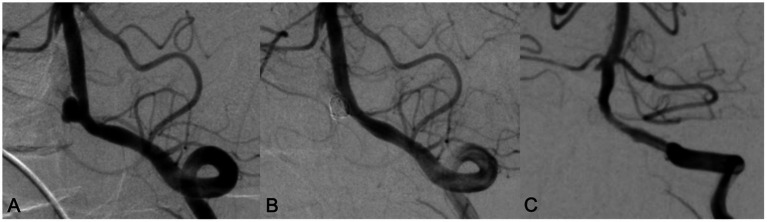
A 70 years old male patient with headache for 5 years. DSA showed a wide neck aneurysm in the V4 segment of the left vertebral artery **(A)**. LVIS stent was used in assisting coil embolization. Immediate angiography showed the aneurysm was completely occluded, with MRRC grading 1 **(B)**. Angiography performed in 10 months’ follow-up showed that the aneurysm was occluded well with MRRC grade 1, but there was mild in-stent stenosis **(C)**.

All patients successfully completed SAC for WNIA, and the technical success rate was 100%. Immediate postembolization angiography showed that in the Neuroform Atlas group, 22 aneurysms (81.5%, 22/27) achieved MRRC Class I, 5 aneurysms (18.5%, 5/27) achieved Class II, and 0 aneurysms (0%) achieved Class III. In the LVIS group, 25 aneurysms (73.5%, 25/34) achieved MRRC Class I, 9 aneurysms (26.5%, 9/34) achieved Class II, and 0 aneurysms (0%) achieved Class III. There was no significant difference in the initial obliteration class between the two groups (*p* = 0.549) (Table 2). Representative cases of the Atlas group and LVIS group were shown in Figures 1, 2, respectively.

**Table 2:** Summary of the obliteration class, complications and follow-up data.

Evaluation indicators	Atlas	LVIS	P value
Initial obliteration class			0.549
MRRC Class I	22 (81.5%)	25 (73.5%)	
MRRC Class II	5 (18.5%)	9 (26.5%)	
MRRC Class III	0 (0%)	0 (0%)	
Complications			
Intraoperative bleeding	0 (0%)	2 (5.9%)	0.501
Intraoperative thrombosis	0 (0%)	1 (2.9%)	1.000
Postoperative ischemic stroke	0 (0%)	0 (0%)	---
mRS score (3 m follow-up)	26 (100%)	34 (100%)	0.438
0	25	31	
1	1	2	
2	0	1	
3	0	0	
4–6	0	0	
Patients with latest DSA follow-up	25 (96.2%)	32 (94.1%)	1.000
Follow-up obliteration class			
MRRC Class I	22 (88%)	28 (87.5%)	0.955
MRRC Class II	3 (12%)	4 (12.5%)	
MRRC Class III	0 (0%)	0 (0%)	

### 3.3 Complications

In the Neuroform Atlas group, a total of 29 stents were used in 26 patients with 27 aneurysms, with no cases of intraoperative cerebral hemorrhage, acute in-stent thrombosis, or postoperative ischemic stroke. In the LVIS group, a total of 34 stents (18 LVIS stents and 16 LVIS Jr. stents) were used in 34 patients with 34 aneurysms. In this group, intraoperative aneurysm rupture occurred in 2 patients (5.9%, 2/34), one of which (2.9%, 1/34) was complicated by acute in-stent thrombosis. No postoperative ischemic strokes occurred. There were no significant differences in the incidence of intraoperative bleeding (*p* = 0.501), intraoperative thrombosis (*p* = 1.000) and postoperative ischemic stroke between the two groups.

### 3.4 Intraoperative complication cases

**Figure 3:**
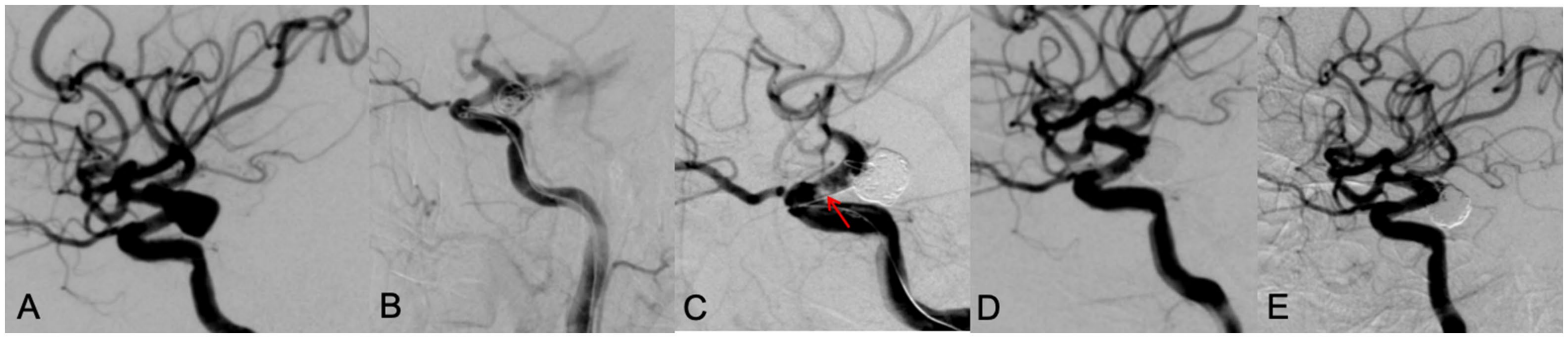
A 65 year old male patient with diplopia for 3 weeks. DSA showed a left posterior communicating aneurysm with wide neck **(A)**. During the LVIS stent assisting embolization, the aneurysm ruptured with contrast leakage **(B)**. After rapid dense coil packing, contrast leakage was stopped, however, an in-stent thrombosis (red arrow) was observed **(C)**. After intravenous use of tirofiban, thrombosis disappeared, the aneurysm was occluded with MRRC grade 1 **(D)**. A 9 months’ follow-up DSA examination showed the aneurysm was occluded well with MRRC grade 1 **(E)**.

Intraoperative complications occurred in two patients, both of them were in the LVIS group. One patient had a left posterior communicating artery aneurysm. During the procedure, the aneurysm ruptured. Immediately, emergent protamine reversal of heparin was applied, and multiple ev3 coils were packed through another microcatheter until angiography showed no contrast extravasation, however, an in-stent thrombosis was noted. Tirofiban was then administered. The final angiography showed patent vessels, disappeared in-stent thrombosis, and occlusion of the aneurysm (Figure 3). The patient recovered well. The second patient had a left vertebral artery wide-neck aneurysm. During LVIS stent-assisted embolization, the aneurysm ruptured. Rapid coil packing was performed, and as contrast extravasation persisted, finally the parent artery and the aneurysm were all occluded. Because of the patent contralateral vertebral artery, the patient recovered well postoperatively.

### 3.5 Follow-up results

**Figure 4:**
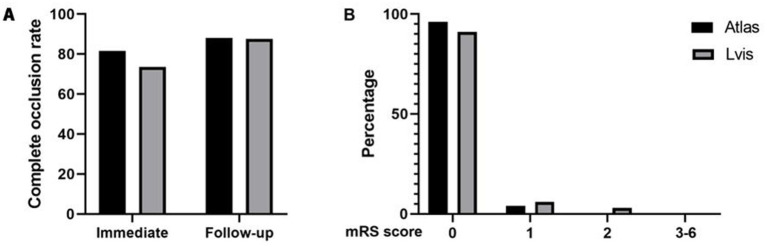
Comparison of complete occlusion rate between two groups of aneurysms immediately after surgery and during follow-up **(A)**. Comparison of mRS scores between two groups of patients at 3-month follow-up after surgery **(B)**.

All 60 patients were followed up regularly via telephone or outpatient visit. At 3 months postoperatively, all patients had mRS scores of 0–2, indicating good neurological outcomes. In the Atlas group, 25 patients had an mRS score of 0 and 1 patient had an mRS score of 1. In the LVIS group, 31 patients had an mRS score of 0, 2 patients had an mRS score of 1, and 1 patient had an mRS score of 2 (Figure 4).

The median time interval from surgery to follow-up was 26 months (Atlas) and 29 months (LVIS). In the Atlas group, 24 patients with 25 aneurysms (92.3%) underwent follow-up DSA examination. Among them, 22 aneurysms (88%, 22/25) were classified as MRRC Class I, and 3 aneurysms (12%, 3/25) were classified as MRRC Class II (one aneurysm with recurrence). In the LVIS group, 32 patients (94.1%) underwent follow-up DSA. Among them, 28 aneurysms (87.5%, 28/32) were classified as Class I, and 4 aneurysms (12.5%, 4/32) were classified as Class II (including one aneurysm with recurrence). There was no significant difference in the follow-up obliteration class between the two groups (*p* = 0.955) (Figure 4).

## 4 Discussion

Our study suggested that both the Neuroform Atlas stent and LVIS stent played an important role in assisting coil embolization of WNIAs and achieved satisfactory immediate and follow-up results. The immediate complete obliteration (MRRC 1) rates of aneurysms were 81.5% (22/27) in the Neuroform Atlas group and 73.5% (25/34) in the LVIS group; with follow-up DSA showing these rates of 88% (22/25) and 87.5% (28/32) respectively. All patients had a favorable clinical outcomes with an mRS score of 0–2 during follow-up. In our study, there was no significant difference in the initial MRRC class, complication rates, and follow-up MRRC class, mRS scores between the Neuroform Atlas stent and the LVIS stent groups.

### 4.1 Comparison with previous studies

At present, there are many types of stents used internationally for assisting coil embolization of aneurysms. In addition to the two stents mentioned in this study, there are Neuroform EZ stent, Enterprise stent, Solitaire stent, Leo stent, and so on (4, 9–13). These stents have shown good characteristics in assisting coil embolization of aneurysms, achieving good embolization effects. Relevant research results show that the mean immediate occlusion rate of SAC is 50.20% (95% CI: 36.09–64.30) in the treatment of unruptured WNIAs, and the occlusion rate of aneurysms is higher in long-term follow-up [mean rate is 63.83% (95% CI: 45.80–80.18)] (14). This study selected the Neuroform Atlas stent and LVIS stent to investigate the effects of different structural designs on the outcomes of assisted embolization of WNIAs.

The Neuroform Atlas stent is an open-cell laser-cut metal intravascular stent produced by Stryker. Atlas stent can be delivered via SL-10 (ID = 0.0165in) or XT-17 (ID = 0.017in) microcatheters (15). The Atlas stent has a low length shortening rate, and the mesh adopts a hybrid ring design with high openness and compliance, which facilitates the free entry and exit of the catheter. The stent also has independent working stent segments with good wall adhesion and deployment ability in tortuous blood vessels (16). LVIS stent is a self-expanding nickel-titanium, monofilament braided closed-cell metal stent manufactured by Microvention, USA, which can be released and retrieved (17). The stent adopts a closed-cell structure, high metal coverage and small pore size providing consistent support of coil mass including small finishing coils. The Atlas stent and the LVIS stent have differences in physical properties. As shown in Table 3, the LVIS stent has higher pore density, mental surface coverage, perpendicular radial force and surface roughness. The circumferential radial force of the Atlas stent is relatively higher. The corrosion resistance of the two stents is comparable (Table 3) (4).

**Table 3:** Comparison of the physical properties of Atlas and LVIS stents [cited from reference Cho et al. (4)].

Physical properties	Atlas stent (4.0 mm diameter, 20.0 mm length).	LVIS stent (4.0 mm diameter, 35.0 mm length).	Difference ratio (LVIS/Atlas)
Structure	Open cell	Closed cell	
Manufacturing process	Laser-cut	Wire-braided	
pore density (pores/mm^2^)	0.276	0.782	283%
Mental surface coverage (%)	10%	23%	230%
perpendicular radial force (gf)	11.4	37.1	325%
Circumferential radial force (gf)	129.8	60.4	47%
Surface roughness (mm)	3.3	13.7	415%
Corrosion resistance (mV)	1,040	1,150	111%

At present, there are many reports about atlas stent and LVIS stent in assisting coil embolization in wide necked intracranial aneurysms (15, 18–29), and most cases have good embolization effect and favorable outcomes, however, the results reported in different literatures are different (Table 4). According to previous literature studies, the complete immediate aneurysm occlusion rate of the Atlas stent in assisting coil embolization is between 45.3–84%, the complete follow-up aneurysm occlusion rate is between 53.8–94.1%, and the complication rate is between 2.1–15.4% (18–25). The complete immediate aneurysm occlusion rate of the LVIS stent is between 25.5–86%, the complete follow-up aneurysm occlusion rate is between 82.1–92.7%, and the complication rate is between 1 and 11.6% (25–29). The immediate aneurysm occlusion rates of the two stents were quite different. The complete follow-up aneurysm occlusion rates of both were high, the complication rates were relatively low, and the differences in efficacy between the two were not obvious. Both showed good safety and effectiveness (15, 18–29).

**Table 4:** Summary of occlusion rates and complications of Atlas and LVIS stent.

Study	Stent type	Study design	No. of patients And aneurysms	Technical success	Immediate aneurysm occlusion rate	Follow-up aneurysm occlusion rate	Complication rate	Mean follow-up period
Cay et al. (18)	Atlas	Retrospective, single center	48 patients (55 aneurysms)	100%	–	MRRC1 or MRRC2 in 94.1%	2.1% (minor stroke)	7.9 months
Ulfert et al. (15)	Atlas	Retrospective, multicenter	36 patients (37 aneurysms)	100%	MRRC1 in 84%, MRRC2 in 17%	MRRC1 in 93%, MRRC2 in 7%	2.8% (one patient with transitory ischaemic attack)	6.1 months
Gross et al. (19)	Atlas	Retrospective, single-center	37 patients (37 aneurysms)	100%	MRRC1 in 57% MRRC2 in 24% MRRC3 in19%	MRRC1 in 92% MRRC2 in 8% MRRC3 in 0%	3% (major stroke)	12.1 months
Quintana et al. (20)	Atlas	Retrospective, single center	29 patients (30aneurysms)	96.7%	MRRC1 in 56.6% MRRC2 in 40% MRRC3 in 3.3%	MRRC1 in 60%	10%, with no permanent deficits	12 months
Ten Brinck et al. (21)	Atlas	Retrospective, single center	27 patients (27 aneurysms)	88.9%	MRRC1 in 63%, MRRC2 in 11.1% MRRC3 in 25.9%	MRRC1 in 53.8% MRRC2 in 15.4% MRRC3 in 30.7%	Intra-procedural thromboembolic complications in 14.8% and intervention-related stroke in 15.4%	7.9 months
Tsai et al. (22)	Atlas	Retrospective, single center	58 patients [58 aneurysms]	100%	MRRC1 in 70.7%, MRRC2 in 20.7% MRRC3 in 8.6%	–	Total in 6.9% Transient thrombosis in 5.2% aneurysm rupture in 1.7%	–
Sweid et al. (23)	Atlas	Retrospective, multi-center	69 aneurysms	95.7%	MRRC1 in 52% MRRC2 in 35% MRRC3 in 13%	MRRC1 in 63% MRRC2 in 34.7% MRRC3 in 6.3%	Asymptomatic major complications in 10.1%	4 months
Daou et al. (24)	Atlas	Retrospective, single center	77 patients	100%	MRRC1 in 81.8% MRRC2 in 18.2%	MRRC1 in 83.7%, MRRC2 in 9.3% MRRC3 in 7%	Total complications in 6.5%	8.7 months
Di et al. (25)	Atlas	Retrospective, single center	56 patients (64 aneurysms)	-	MRRC1 in 45.3%, MRRC2 in 14.1% MRRC3 in 40.6%	MRRC1 in 75.0% MRRC2 in 10.9% MRRC3 in 14.1%	5.4%	10 months
Liu et al. (26)	LVIS	Retrospective, single center	43patients (43 aneurysms)	-	MRRC1 in 86% MRRC2 in 11% MRRC3 in 2.3%	-	11.6% (Vasospasm 2.3%, Acute embolism 4.7%, Intraoperative bleeding 4.7%)	-
Kang et al. (27)	LVIS	Retrospective, single center	55 patients (55 aneurysms)	100%	MRRC1 in 25.5% MRRC2 in 74.5% MRRC3 in 0%	MRRC1 in 82.1% MRRC2 in 10.3% MRRC3 in 7.7%	7.3% (Stent thrombosis 1.8%, Vasospasm 1.8%, Plaque detachment with cerebral infarction 1.8%Cerebral hemorrhage 1.8%)	6 months
Di et al. (25)	LVIS	Retrospective, single center	45 patients (54 aneurysms)	-	MRRC1 in 64.8% MRRC2 in 14.8% MRRC3 in 20.4%	MRRC1 in 85.2% MRRC2 in5.6% MRRC3 in9.3%	6.7%	10 months
Feng et al. (28)	LVIS	Retrospective, single center	97 patients (107 aneurysms)	100%	MRRC1 in 28.9% MRRC2 in 40.2% MRRC3 in 30.9%	MRRC1 in 84.2% MRRC2 in 11.8% MRRC3 in 4%	1% (acute in-stent thrombosis)	8.1 months
Xue et al. (29)	LVIS	Retrospective	142 patients (aneurysms)	-	MRRC1 in 64.1% MRRC2 in 14.8% MRRC3 in 21.1%	Complete occlusion in 92.7%	5.6%	529 days

MRRC, modified Raymond-Roy Classification.

### 4.2 Interpretation of results

In our study, we found that the immediate aneurysm occlusion rates between the two groups were not statistically different, but the proportion of MRRC grade 1 in the atlas group was 81.5% (22/27), which was slightly higher than 73.5% (25/34) in the LVIS stent group. However, the proportion of MRRC grade 1 patients in the follow-up of the two groups was similar, 88% (22/25) and 87.5% (28/32), respectively. This might be because that with the extension of follow-up time and the reduction of the use of antiplatelet drugs, the blood flow in the aneurysm was stagnant, and the aneurysm neck and sac were finally filled with thrombus. Another reason may be the dense mesh of LVIS stent, in literature, it was hypothesized that LVIS stent might have potential flow diversion properties, and there were also reports that recurrent aneurysms were finally cured by using multiple LVIS stents (30).

### 4.3 Complications

Premature rupture during embolization is a common but severe complication. Previous studies have shown that excessive anticoagulation or antiplatelet therapy before surgery is a risk factor for bleeding (31). Standardized preoperative preparation is particularly important. In-stent thrombosis is another common complication. Patients undergoing elective surgery using SAC for intracranial aneurysm should be given adequate antiplatelet therapy for a sufficient course of treatment before surgery. For patients undergoing emergency surgery, doctors should give intravenous antiplatelet drugs as soon as possible after stent release. Tirofiban is a small molecule inhibitory drug that acts on the protein–protein interaction between fibrinogen and platelet integrin receptor GP IIb/IIIa. A clinical study found that tirofiban showed good safety and efficacy in the preventive use before SAC and after thromboembolic events in patients with intracranial aneurysms (32). In our study, two patients suffered premature rupture during operation, and one of these two patients had in-stent thrombosis. Both patients were in the LVIS stent group. The reasons for its premature rupture may be related to the large aneurysm size, the thin wall, the limited mobility of the microcatheter’s tip in the aneurysm after the release of LVIS stent, and the slightly larger size of the selected coil. After the premature rupture of the aneurysm, the catastrophic outcome can be rescued by neutralizing heparin, rapidly packing the coil, tightly packing the rupture site and the aneurysm neck, or occluding the parent artery if sufficient collateral compensation exists.

### 4.4 Limitations

Although our study suggested that both stents achieved similar satisfactory embolization effects and good prognosis in assisting coil embolization of WNIAs, there were still many limitations in our study: (1) This study is only a single center, small sample retrospective study, and all surgical procedures were performed by a single surgical team. Therefore, there may be a potential operator bias and a selection bias in patient screening, surgical experience, intraoperative stent selection, and use of microcatheters, which may affect the final results of the study. The small sample size will lead to an associated risk of a Type II error (i.e., inadequately powered to detect small differences). (2) The location of aneurysms in Atlas stent group and LVIS stent group is slightly different. For aneurysms with large differences in the distal and proximal diameters of the parent artery, such as those located at the tip of the basilar artery, the bifurcation of the internal carotid artery, or the anterior choroidal artery, because it is not suitable for LVIS stent, we usually use atlas stent in such patients. For some WNIAs need to be treated with Y shaped-stents, we also rarely use LVIS stents. All these above undermines random comparability. However, the number of such patients was low, and it does not affect the statistical results. (3) The trends toward differences in baseline characteristics between the Atlas and LVIS groups regarding age (*p* = 0.068) and history of hypertension (*p* = 0.063) seems near-significant imbalances which might confound outcomes. However, in our study, a 5-year difference (67.7 vs. 62.5 years) and a history of hypertension may be not typically considered a decisive factor affecting technical success, immediate packing density, or long-term aneurysm occlusion in neurointerventional practice. The two groups were well-matched on crucial baseline metrics that more directly influence treatment strategy and outcomes, such as aneurysm size, location, and rupture status. This considerably reduces the likelihood of age and hypertension acting as major confounders. (4) In this study, unruptured aneurysms accounted for the majority, while ruptured aneurysms were rare, and the Hunt Hess grading was low, which cannot fully reflect the true clinical status. (5) Although the number of patients using dual microcatheter technology for embolization in both groups was not high, the proportion of Atlas group patients using dual microcatheter for aneurysm filling was slightly higher than that of LVIS group, which was determined by the structure of the stent itself and the density of the mesh. The proportion of dual microcatheters used may have an impact on the degree of aneurysm occlusion, leading to biased research results. (6) In this study, although we tried to follow up with patients as much as possible, there were still several patients who were lost to follow-up, and these lost cases may bias the results. (7) This study is a retrospective study, and the confirmation of the research results requires large-scale, multicenter prospective clinical randomized controlled trials.

### 4.5 Future directions

The endovascular treatment of aneurysms is becoming increasingly mature with the development of the interventional materials. With the application of flow diverter, web, etc., the treatment diversity of aneurysms has been enriched, and the treatment effect is also getting better and better. Although the proportion of flow diverter in the treatment of intracranial wide necked aneurysms will continue to increase in the future, it cannot be denied that the stent assisted coil technology cannot be completely replaced. Proper stent selection and reasonable filling techniques will continue to contribute to the treatment of aneurysms.

## 5 Conclusion

In our single-center retrospective exploratory study, both Neuroform Atlas stent and LVIS stent seem to perform good safety and efficacy in assisting coil embolization of WNIAs, however, further multicenter randomized controlled studies with larger patient series and a longer follow up period will be helpful in elucidation of both the efficacy and the longevity.

## Data Availability

The raw data supporting the conclusions of this article will be made available by the authors, without undue reservation.

## References

[1] Shao MM White TG Bassett JB Dowlati E Mehta SH Werner C. Intrasaccular treatment of intracranial aneurysms: a comprehensive review. J Clin Med. (2024) 13:6162. doi: 10.3390/jcm13206162, PMID: 39458111 PMC11508718

[2] Gaub M Murtha G Lafuente M Webb M Luo A Birnbaum LA. Flow diversion for endovascular treatment of intracranial aneurysms: past, present, and future directions. J Clin Med. (2024) 13:4167. doi: 10.3390/jcm13144167, PMID: 39064207 PMC11278297

[3] Higashida RT Smith W Gress D Urwin R Dowd CF Balousek PA. Intravascular stent and endovascular coil placement for a ruptured fusiform aneurysm of the basilar artery. Case report and review of the literature. J Neurosurg. (1997) 87:944–9. doi: 10.3171/jns.1997.87.6.0944, PMID: 9384409

[4] Cho SH Jo WI Jo YE Kim HS Park MS Kim BM. Bench-top comparison of physical properties of 4 commercially-available self-expanding intracranial stents. Neurointervention. (2017) 12:31–9. doi: 10.5469/neuroint.2017.12.1.31 28316867 PMC5355459

[5] Dhar S Tremmel M Mocco J Shojima M Connolly ES Jr Siddiqui AH. Morphology parameters for intracranial aneurysm rupture risk assessment. Neurosurgery. (2008) 63:185–96. doi: 10.1227/01.NEU.0000316847.64140.81 18797347 PMC2570753

[6] Luo A Mascitelli J Birnbaum L Kallmes D Lanzino G Fiorella D. Y-stent-assisted coiling for large wide-neck dysplastic middle cerebral artery bifurcation aneurysm: an update to procedural technique. Surg Neurol Int. (2025) 16:71. doi: 10.25259/SNI_877_2024, PMID: 40041045 PMC11878741

[7] Phyo WSY Shirakawa M Matsukawa H Kuroda S Horiuchi T Tanaka R. The efficacy and safety of stent-assisted coil embolization with the semi-jailing technique in patients with unruptured intracranial aneurysm. J Cerebrovasc Endovasc Neurosurg. (2025). doi: 10.7461/jcen.2025.E2025.01.003, PMID: 40660890 PMC12488327

[8] Mascitelli JR Moyle H Oermann EK Lawton MT Nakaji P Zabramski JM. An update to the Raymond-Roy occlusion classification of intracranial aneurysms treated with coil embolization. J Neurointerv Surg. (2015) 7:496–502. doi: 10.1136/neurintsurg-2014-011258 24898735

[9] Khattak YJ Sibaie AA Anwar M Siddiqui AH Levy EI. Stents and stent mimickers in endovascular management of wide-neck intracranial aneurysms. Cureus. (2018) 10:e3420. doi: 10.7759/cureus.3420, PMID: 30542634 PMC6284878

[10] You W Feng J Ge H Li Y Zhang Y Wang L. Bifurcated aneurysm location predicts in-stent stenosis after Neuroform-EZ stent-assisted coiling for intracranial aneurysm. Front Neurol. (2022) 13:873014. doi: 10.3389/fneur.2022.873014 35645959 PMC9136285

[11] Wu D Lai N Zhao X Lin L Chen J Ye X. Enterprise 2 stent-assisted embolization of paraclinoid aneurysms: a single center preliminary study. Clin Interv Aging. (2022) 17:1833–40. doi: 10.2147/CIA.S390882 36536798 PMC9759008

[12] Junior ZD Alencar G Koppe GL de Souza AC de Carvalho FB de Mello RG. Solitaire AB stent deployment for treatment of basilar apex aneurysm via the posterior communicating artery. Turk Neurosurg. (2022) 32:517–20. doi: 10.5137/1019-5149.JTN.34847-21.3, PMID: 35253150

[13] Phuyal S Lamichhane S Mishra B Baral S Karki S Rijal S. "shelf technique" in braided stent (Leo baby) in wide-necked intracranial aneurysm. J Neurosci Rural Pract. (2023) 14:528–30. doi: 10.25259/JNRP_23_2023, PMID: 37692819 PMC10483209

[14] Papadopoulos F Antonopoulos CN Geroulakos G. Stent-assisted coiling of unruptured intracranial aneurysms with wide neck. Asian J Neurosurg. (2020) 15:821–7. doi: 10.4103/ajns.AJNS_57_20, PMID: 33708649 PMC7869257

[15] Ulfert C Pham M Sonnberger M Gizewski ER Sure U Berkefeld J. The neuroform atlas stent to assist coil embolization of intracranial aneurysms: a multicentre experience. J Neurointerv Surg. (2018) 10:1192–6. doi: 10.1136/neurintsurg-2017-013516 29678886

[16] Caragliano AA Papa R Pitrone A Cognard C Pierot L Spelle L. The low-profile Neuroform atlas stent in the treatment of wide-necked intracranial aneurysms - immediate and midterm results: an Italian multicenter registry. J Neuroradiol. (2020) 47:421–7. doi: 10.1016/j.neurad.2019.03.005 30951769

[17] Shi S Long S Hui F Zhang Y Wang Y Li J. Safety and efficacy of LVIS Jr stent-assisted coiling of intracranial aneurysms in small-diameter parent arteries: a single-center experience. Clin Neuroradiol. (2024) 34:587–95. doi: 10.1007/s00062-024-01397-0, PMID: 38451269

[18] Cay F Peker A Arat A. Stent-assisted coiling of cerebral aneurysms with the Neuroform atlas stent. Interv Neuroradiol. (2018) 24:263–9. doi: 10.1177/1591019917753710, PMID: 29350091 PMC5967178

[19] Gross BA Ares WJ Ducruet AF Albuquerque FC Gonzalez LF Tjoumakaris SI. A clinical comparison of atlas and LVIS Jr stent-assisted aneurysm coiling. J Neurointerv Surg. (2019) 11:171–4. doi: 10.1136/neurintsurg-2018-014208 30077966

[20] Quintana EM Valdes PV Deza EM Roa JA Sosa AR Biondi A. Initial experience and one-year follow-up with Neuroform atlas stent system for the treatment of brain aneurysms. Interv Neuroradiol. (2019) 25:521–9. doi: 10.1177/1591019918819087 30939955 PMC6777111

[21] Ten Brinck MFM de Vries J Bartels RHMA Sluzewski M Van der Schaaf IC Van Rooij WJ. NeuroForm atlas stent-assisted coiling: preliminary results. Neurosurgery. (2019) 84:179–89. doi: 10.1093/neuros/nyy048, PMID: 29579261

[22] Tsai JP Hardman J Moore NZ Hanel RA Albuquerque FC Halbach VV. Early post-humanitarian device exemption experience with the Neuroform atlas stent. J Neurointerv Surg. (2019) 11:1141–4. doi: 10.1136/neurintsurg-2019-014874, PMID: 30979847

[23] Sweid A Herial N Sajja K Hasan D Tjoumakaris SI Rosenwasser RH. Early multicenter experience with the Neuroform atlas stent: feasibility, safety, and efficacy. Neurosurgery. (2020) 87:E321–35. doi: 10.1093/neuros/nyaa143, PMID: 32453816

[24] Daou BJ Palmateer G Linzey JR Dion JE Tong FC Grossberg JA. Stent-assisted coiling of cerebral aneurysms: head to head comparison between the Neuroform atlas and EZ stents. Interv Neuroradiol. (2021) 27:353–61. doi: 10.1177/1591019921989476, PMID: 33509014 PMC8190941

[25] Di RY Ge L Lu G Shen L Yu Z Xu J. Clinical and angiographic outcomes of stent-assisted coiling of paraclinoid aneurysms: comparison of LVIS and Neuroform stents. J Clin Neurosci. (2021) 83:1–7. doi: 10.1016/j.jocn.2020.11.047, PMID: 33341366

[26] Liu Y Sun Y He Y Zhang L Wang X Li H. Treatment efficacy of solitaire stent- and LVIS stent-assisted coil embolization for intracranial wide-neck carotid aneurysm. Nan Fang Yi Ke Da Xue Xue Bao. (2020) 40:423–6. doi: 10.12122/j.issn.1673-4254.2020.03.23 32376577 PMC7167311

[27] Kang J Tian X Wu Q Zhang H Li Y Wang Z. LVIS stent-assisted coil embolization in the acute stage of ruptured intracranial aneurysms. Zhonghua Wei Zhong Bing Ji Jiu Yi Xue. (2020) 32:828–34. doi: 10.3760/cma.j.cn121430-20200514-00389 32788018

[28] Feng Z Fang Y Xu Y Yan Y He X Lv G. The safety and efficacy of low profile visualized intraluminal support (LVIS) stents in assisting coil embolization of intracranial saccular aneurysms: a single center experience. J Neurointerv Surg. (2016) 8:1192–6. doi: 10.1136/neurintsurg-2015-012090, PMID: 26747876

[29] Xue G Zuo Q Zhang X Wang Y Li J Liu C. Safety and efficacy of stent-assisted coiling for acutely ruptured wide-necked intracranial aneurysms: comparison of LVIS stents with laser-cut stents. Chin Neurosurg J. (2021) 7:19. doi: 10.1186/s41016-021-00237-1, PMID: 33653398 PMC7927374

[30] Suzuki R Takigawa T Anazawa T Tanaka Y Yamaguchi S Oka H. A patient with a ruptured blood blister-like aneurysm of the internal carotid artery in whom LVIS stents were inserted. J Neuroendovasc Ther. (2020) 14:102–7. doi: 10.5797/jnet.cr.2019-0068 37502387 PMC10370645

[31] Serebruany VL Steinhubl SR Berger PB. Analysis of risk of bleeding complications after different doses of aspirin in 192,036 patients enrolled in 31 randomized controlled trials. Am J Cardiol. (2005) 95:1218–22. doi: 10.1016/j.amjcard.2005.01.049, PMID: 15877994

[32] Yan Y He X Fang Y Feng Z Lv G Xu Y. The safety and efficacy of low-dosage tirofiban for stent-assisted coiling of ruptured intracranial aneurysms. Neurosurg Rev. (2021) 44:2211–8. doi: 10.1007/s10143-020-01398-w 32989479

